# Machine Learning Algorithms to Predict Venous Thromboembolism in Patients With Sepsis in the Intensive Care Unit: Multicenter Retrospective Study

**DOI:** 10.2196/80969

**Published:** 2026-01-30

**Authors:** Yan Zhang, Xia Ren, Luojie Liu, Junjie Zha, Yijie Gu, Hongwei Ye

**Affiliations:** 1 Department of Emergency and Critical Care Medicine Changshu No.1 People's Hospital Changshu, Jiangsu China; 2 Department of Gastroenterology Changshu No.1 People's Hospital Changshu, Jiangsu China

**Keywords:** venous thromboembolism, sepsis, machine learning, SHAP, MIMIC-IV

## Abstract

**Background:**

Venous thromboembolism (VTE) is a common and severe complication in intensive care unit (ICU) patients with sepsis. Conventional risk stratification tools lack sepsis-specific features and may inadequately capture complex, nonlinear interactions among clinical variables.

**Objective:**

This study aimed to develop and validate an interpretable machine learning (ML) model for the early prediction of VTE in ICU patients with sepsis.

**Methods:**

This multicenter retrospective study used data from the Medical Information Mart for Intensive Care IV database for model development and internal validation, and an independent cohort from Changshu Hospital for external validation. Candidate predictors were selected through univariate analysis, followed by least absolute shrinkage and selection operator regression. Retained variables were used in multivariable logistic regression to identify independent predictors, which were then used to develop 9 ML models, including categorical boosting, decision tree, k-nearest neighbor, light gradient boosting machine, logistic regression, multilayer perceptron, naive Bayes, random forest, and support vector machine. Performance was evaluated by discrimination (area under the curve [AUC]), calibration, and clinical use (decision curve analysis). A subgroup analysis stratified by the Sequential Organ Failure Assessment score was conducted in the external cohort to assess model stability across sepsis severity levels. Model interpretability was assessed using Shapley Additive Explanations (SHAP) to quantify the contribution of features to the predicted risk.

**Results:**

A total of 25,197 patients from the Medical Information Mart for Intensive Care IV cohort and 328 patients from the external cohort were included, with VTE incidences of 844 out of 25,197 (3.4%) and 30 out of 328 (9.2%), respectively. The light gradient boosting machine model performed best, achieving an AUC of 0.956 in internal validation. Despite the higher VTE incidence and clinical severity in the external validation, the model maintained robust generalization with an AUC of 0.786. Notably, the model achieved enhanced discriminative ability in the severe sepsis subgroup (Sequential Organ Failure Assessment score >6) with an AUC of 0.816, compared with 0.769 in the mild to moderate sepsis subgroup. Calibration curves indicated strong agreement between predicted and observed outcomes, and decision curve analysis showed superior net benefit across clinically relevant thresholds. SHAP analysis identified central venous catheterization, serum chloride and bicarbonate levels, arterial catheterization, and prolonged partial thromboplastin time as the most influential predictors. Partial dependence plots revealed both linear and nonlinear associations between these variables and VTE risk. Individual-level force plots further enhanced interpretability by visualizing personalized risk profiles.

**Conclusions:**

We developed a high-performing and interpretable ML model for predicting VTE in ICU patients with sepsis. The model demonstrated robustness across cohorts and enhanced performance in the severe sepsis population. By integrating diverse clinical data and leveraging SHAP for transparent explanations, this tool may support personalized prophylaxis and early diagnostic strategies.

## Introduction

Sepsis is a life-threatening condition characterized by acute organ dysfunction resulting from a dysregulated host response to infection and remains a leading cause of mortality in the intensive care unit (ICU) [1]. According to the Global Burden of Disease Study, approximately 48.9 million cases of sepsis occur annually worldwide, resulting in an estimated 11.0 million deaths, which accounts for 19.7% of all global deaths [2]. Venous thromboembolism (VTE), comprising deep vein thrombosis and pulmonary embolism (PE), is a common and severe complication of sepsis that significantly worsens patient outcomes. Despite adherence to guideline-recommended thromboprophylaxis, the incidence of VTE in patients with sepsis remains high at 37.2%, up to 10-fold greater than in ICU populations with no sepsis [3]. Previous studies have shown that the presence of VTE is associated with a 28-day mortality rate of 28.6% among patients with sepsis, and the occurrence of PE nearly doubles the risk of in-hospital death (odds ratio [OR] 1.94) [4,5]. These findings underscore the urgent need for early and accurate VTE risk stratification in sepsis to guide timely clinical interventions and reduce morbidity and mortality in this high-risk population.

Current clinical methods for predicting VTE in patients with sepsis have notable limitations. Traditional biomarkers such as D-dimer, although widely used, lack specificity in the context of sepsis, as the condition itself activates coagulation pathways and elevates fibrin degradation products [6,7]. Furthermore, commonly used scoring systems such as the Wells and Autar scores were primarily developed and validated in trauma or postoperative populations. These tools do not account for sepsis-specific indicators, such as the Sequential Organ Failure Assessment (SOFA) score or lactate levels, thereby limiting their predictive use in patients with sepsis [8-10]. Although multivariable logistic regression (LR) models that incorporate clinical and laboratory variables have shown promise, with reported area under the curve (AUC) values reaching up to 0.87, these models are inherently linear and may fail to capture the complex and nonlinear interactions among sepsis-related risk factors [11,12].

Machine learning (ML) techniques have emerged as powerful tools for handling high-dimensional data, uncovering nonlinear associations, and automating feature selection. In recent years, ML models have demonstrated superior performance in predicting complications such as septic shock and acute kidney injury (AKI) in ICU patients, with reported AUC values reaching up to 0.90 [13-15]. However, earlier generations of ML models often functioned as “black boxes,” which limited their adoption in clinical settings due to the lack of interpretability. Explainable ML approaches, such as Shapley Additive Explanations (SHAP), have been developed to address this challenge. By quantifying the contribution of each feature, SHAP enhances model transparency and facilitates clinical understanding [16]. For instance, Liu et al [17] developed an interpretable ML model for septic shock prediction, whose explanatory outputs aligned closely with hemodynamic mechanisms described in the Sepsis-3 guidelines, thereby reinforcing its clinical relevance [18].

Therefore, this study aimed to develop an interpretable ML model that integrates clinical features and laboratory indicators to predict the risk of VTE in ICU patients with sepsis. By applying SHAP to interpret model outputs and identify key predictors, we sought to create a decision support tool that combines high predictive accuracy with strong clinical interpretability, ultimately facilitating personalized prevention strategies.

## Methods

### Data Sources

The training and internal validation datasets were extracted from the Medical Information Mart for Intensive Care IV version 3.0 (MIMIC-IV v3.0) database, which contains comprehensive and high-quality data from 65,366 patients admitted to the ICU at Beth Israel Deaconess Medical Center between 2008 and 2022. One of the authors (YZ) completed the Collaborative Institutional Training Initiative examination (record ID: 60227322) and was granted access to the database for research purposes.

### Ethical Considerations

Ethical approval for the use of MIMIC-IV data was granted by the institutional review boards of Beth Israel Deaconess Medical Center and the Massachusetts Institute of Technology, with a waiver of informed consent due to the use of deidentified data. The data were handled in accordance with the Health Insurance Portability and Accountability Act standards to ensure patient privacy and confidentiality. In addition, ICU patients admitted to Changshu Hospital affiliated to Soochow University between January 2019 and August 2024 were included as an external validation cohort. Ethical approval for this component of the study was obtained from the ethics review committee of Changshu Hospital (number L2024055). As the study was observational in nature and used routinely collected clinical data, the requirement for written informed consent was waived. All electronic data were anonymized before analysis, and access to the dataset was restricted to the primary research team to maintain strict confidentiality. All procedures conformed to the ethical standards set forth in the Declaration of Helsinki. No compensation was provided to any participants involved in this study.

### Study Population

This study included adult ICU patients (aged 18 years and older) with sepsis. For patients with multiple ICU admissions, only the first admission was analyzed. Sepsis was defined according to the Third International Consensus Definitions for Sepsis and Septic Shock (Sepsis-3), which require a suspected or confirmed infection accompanied by an increase in the SOFA score of 2 points or more [18]. VTE was defined as an acute episode of deep vein thrombosis (either upper or lower extremities), superficial vein thrombosis (either upper or lower extremities), or PE. VTE diagnoses were identified based on either the *ICD-9* (*International Classification of Diseases, Ninth Revision*; codes 45119, 4512, 45181, 45182, 45183, 45184, 45189, 4519, 4532, 4538, 45381, 45382, 45383, 45384, 45385, 45386, 45387, 45389, 4539, 4150, 41511, 41512, 41513, 41519, 45340, 45341, 45342, 4510, 452, 4530, 4531, and 4533) or the *ICD-10* (*International Statistical Classification of Diseases, Tenth Revision*; codes I808, I809, I8290, I82890, I2699, I2692, I2690, I2602, I2609, I8000, I8001, I8002, I81, I820, and I821) in the MIMIC-IV database, or through diagnostic imaging reports from Changshu Hospital, including duplex venous ultrasonography and contrast-enhanced computed tomography. In the Changshu Hospital cohort, the ICU implemented a routine lower limb venous ultrasound screening protocol for all patients with sepsis meeting high-risk criteria. Patients were excluded meeting any of the following criteria: (1) ICU stay of less than 24 hours, (2) a documented VTE diagnosis prior to sepsis onset, and (3) VTE events occurring within 24 hours following the sepsis diagnosis.

**Figure 1:**
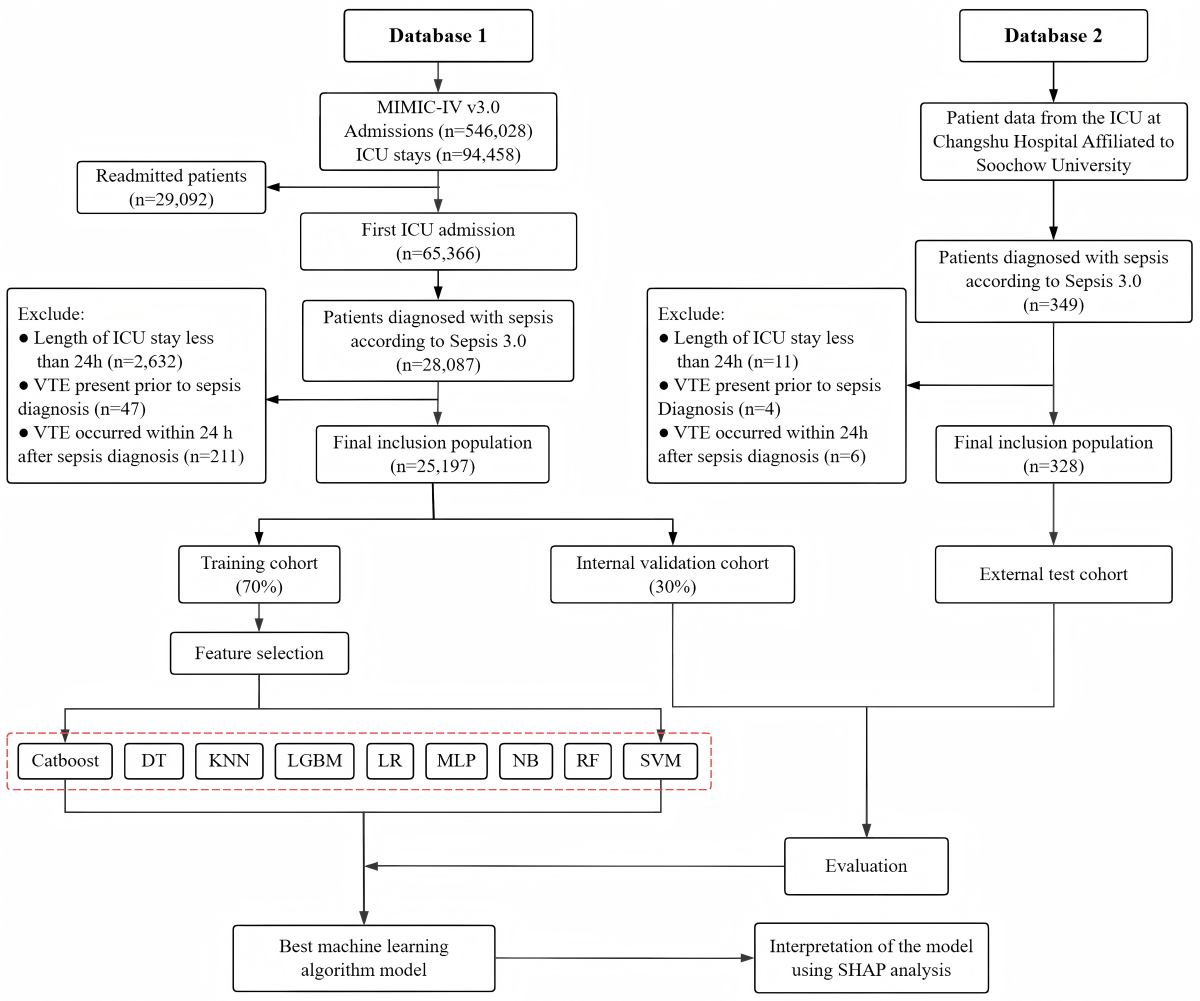
Flowchart of patient enrollment and cohort selection. CatBoost: categorical boosting; DT: decision tree; ICU: intensive care unit; KNN: k-nearest neighbor; LGBM: light gradient boosting machine; LR: logistic regression; MIMIC: Medical Information Mart for Intensive Care Unit; MLP: multilayer perceptron; NB: naive Bayes; RF: random forest; SHAP: SHapley Additive exPlanations; SVM: support vector machine; VTE: venous thromboembolism.

Patients from the MIMIC-IV cohort were randomly split into a training set and an internal validation set in a 7:3 ratio. The training set was used for variable selection and model development, while the internal and external validation sets were used to assess model performance. The overall study workflow is illustrated in Figure 1.

### Data Extraction and Processing

Data from the MIMIC-IV database were extracted using Structured Query Language via pgAdmin 4 (version 6.21; pgAdmin Development Team). All clinical and laboratory variables were collected within the first 24 hours of ICU admission, with only the initial measurement included for variables with multiple readings. A set of variables was extracted for each patient, encompassing (1) demographics: age (years), sex, race (Asian, Black, White, Hispanic, and other), height (cm), weight (kg), BMI (kg/m^2^), and insurance (Medicare or other); (2) vital signs: heart rate (HR, times per minute), respiratory rate (RR, times per minute), systolic blood pressure (mm Hg), diastolic blood pressure (mm Hg), temperature (°C), and oxygen saturation (SpO_2_, %); (3) severity scores: Glasgow Coma Scale, SOFA score, and simplified acute physiology score II (SAPS II); (4) laboratory data: white blood cell count (K/‌μL), hemoglobin (g/dL), platelet (PLT, K/μL), C-reactive protein (mg/dL), alanine aminotransferase (U/L), aspartate aminotransferase (U/L), albumin (g/dL), creatinine (mg/dL), sodium (mEq/L), potassium (mEq/L), chloride (mEq/L), calcium (mg/dL), blood urea nitrogen (mg/dL), hematocrit (%), glucose (mg/dL), international normalized ratio, prothrombin time (PT, seconds), partial thromboplastin time (PTT, seconds), pH, partial pressure of oxygen (PaO_2_, mm Hg), partial pressure of carbon dioxide (PaCO_2_, mm Hg), base excess (mEq/L), lactic acid (mEq/L), and bicarbonate (HCO_3_^-^, mEq/L); (5) ventilation parameters: positive end-expiratory pressure (PEEP, cmH₂O), fraction of inspired oxygen (FiO₂, %), and PEEP/FiO₂ ratio; (6) comorbidities: hypertension, diabetes, myocardial infarction (MI), chronic obstructive pulmonary disease (COPD), asthma, pancreatitis, acute respiratory failure (ARF), AKI, acute respiratory distress syndrome (ARDS), and malignant cancer; and (7) therapeutic interventions: arterial catheterization, central venous catheterization, cardiopulmonary bypass, continuous renal replacement therapy (CRRT), ventilation, heparin, aspirin, and vasopressors use. The same set of variables was extracted from the external validation cohort at Changshu Hospital affiliated with Soochow University, ensuring consistency between the datasets.

For missing data, variables with less than 5% missingness were imputed using mean substitution. For those with 5%-30% missing values, multiple imputation methods were used. Variables with more than 30% missing data were excluded from analysis to minimize bias and preserve model robustness. Outliers were identified and treated as missing values and subsequently handled using the same imputation strategies.

### Statistical Analysis

All statistical analyses were performed using Stata software (version 16.0; StataCorp LLC), R software (version 4.1.2; R Foundation for Statistical Computing), DecisionLinnc (version 1.0; DecisionLinnc Corp), and Python (version 3.9.12; Python Software Foundation). The Kolmogorov-Smirnov test was used to assess the normality of continuous variables. Variables with a normal distribution were expressed as means and SDs, and group comparisons were conducted using the 2-tailed independent samples *t* test. Non–normally distributed variables were presented as median and IQR and analyzed using the Mann-Whitney *U* test. Categorical variables were summarized as counts (percentages) and compared using the chi-square test. Two-sided *P* values of <.05 were considered statistically significant.

### Variable Selection and Model Development

Variables that showed statistically significant differences in the univariate analysis were considered candidate predictors. These variables were further refined using the least absolute shrinkage and selection operator regression to minimize overfitting and reduce multicollinearity. Predictors with nonzero coefficients were subsequently entered into a multivariate LR model. Independent predictors identified through this process were incorporated into the ML models.

Following variable selection, the Synthetic Minority Oversampling Technique (SMOTE) preprocessing algorithm was applied to address class imbalance between VTE and non-VTE cases in the training cohort. In the training cohort, VTE cases (576/17,637, 3.27%) were highly unbalanced compared with non-VTE cases (17,061/17,637, 96.73%). After SMOTE application, the VTE class was balanced to match the non-VTE class, resulting in a 50:50 distribution with a total of 34,122 samples used for model training. This oversampling method helped ensure that the models were trained on a balanced dataset, thereby improving their ability to detect minority class events. A total of 9 ML algorithms were trained on the development cohort, including categorical boosting (CatBoost), decision tree, k-nearest neighbor, light gradient boosting machine (LGBM), LR, multilayer perceptron, naive Bayes, random forest (RF), and support vector machine. For all ML models, we used a 5-fold cross-validation strategy, combined with Grid Search, for hyperparameter optimization and model selection. Notably, SMOTE was applied exclusively to the training folds of the cross-validation process to prevent data leakage into the validation folds or the internal validation set.

### Model Evaluation and Interpretation

Model performance was assessed using the internal and external validation datasets. Discrimination was evaluated by plotting receiver operating characteristic curves and calculating the AUC. Calibration curves were used to compare predicted probabilities with observed outcomes. Clinical use was assessed using decision curve analysis, which estimated the net benefit across a range of probability thresholds. In addition, several evaluation metrics were computed, including accuracy, sensitivity, specificity, and the Youden index, to offer a comprehensive assessment of model performance.

The best performing model was selected based on comprehensive evaluation of these metrics. To assess the model’s stability and performance in different risk populations, a stratified analysis was conducted on the external validation cohort based on the SOFA score. Patients were divided into 2 subgroups: mild to moderate sepsis (SOFA score ≤6) and severe sepsis (SOFA score >6). To enhance model interpretability, SHAP analysis was conducted. SHAP values were computed to quantify the contribution of individual features to the model’s predictions and to explore feature interactions. Visualization tools from the SHAP package in Python, including feature importance plots, bee swarm plots, force plots, and partial dependence plots (PDPs), were used to provide an intuitive understanding of the model’s decision-making process.

## Results

### Baseline Characteristics

A total of 25,197 patients from the MIMIC-IV cohort and 328 patients from the external validation cohort were included in the study. Among them, 844 out of 25,197 (3.4%) patients in the MIMIC-IV cohort and 30 out of 328 (9.2%) patients in the external cohort developed VTE. The baseline characteristics of all patients are summarized in Table 1. In the MIMIC-IV cohort, patients who developed VTE were younger and exhibited significantly higher HRs and RRs than those with no VTE (all *P*<.001). Laboratory parameters revealed higher PLT counts, serum chloride levels, and PTT in the VTE group (all *P*<.05). In addition, patients with VTE had higher illness severity scores, as indicated by elevated SOFA and SAPS II scores (both *P*<.05). Comorbidities such as ARF, AKI, and ARDS were significantly more common in the VTE group (all *P*<.001). Furthermore, these patients were more likely to have undergone invasive procedures, including arterial and central venous catheterization (both *P*<.001). In the external validation cohort, similar patterns were observed. Patients with VTE demonstrated higher rates of ARDS and more frequent use of mechanical ventilation than patients with no VTE (both *P*<.001). These findings highlight significant differences in demographics, clinical severity, comorbidities, and therapeutic interventions between patients with and with no VTE across both cohorts, emphasizing the relevance of these factors in VTE risk stratification.

**Table 1:** Baseline characteristics of the MIMIC-IV^a^ and external validation cohorts.

Variables					MIMIC-IV cohort (N=25,197)										External Validation cohort (N=328)						
					Non-VTE^b^ (n=24,353)			VTE (n=844)			*P* value			Non-VTE (n=298)				VTE (n=30)			*P* value
**Demographic data**																					
	Age (years), mean (SD)			66.61 (16.28)			63.83 (16.75)			<.001			73.33 (12.60)				75.90 (9.88)			.19	
	Sex (male), n (%)			14173 (58.2)			502 (59.5)			.46			210 (70.5)				22 (73)			.74	
	BMI, mean (SD)			27.71 (6.20)			28.06 (6.15)			.11			22.78 (2.59)				21.93 (3.45)			.20	
	**Race, n (%)**			N/A^c^			N/A			.14			N/A				N/A			N/A	
		Asian	1994 (8.2)			80 (9.5)			N/A			N/A				N/A			N/A		
		Black	15812 (64.9)			568 (67.3)			N/A			N/A				N/A			N/A		
		White	806 (3.3)			27 (3.2)			N/A			N/A				N/A			N/A		
		Hispanic	5038 (20.7)			147 (17.4)			N/A			N/A				N/A			N/A		
		Other	703 (2.9)			22 (2.6)			N/A			N/A				N/A			N/A		
	**Insurance, n (%)**			N/A			N/A			.02			N/A				N/A			.09	
		Other	4,027 (16.5)			166 (19.7)			N/A			83 (27.9)				4 (13)			N/A		
		Medicare	20326 (83.5)			678 (80.3)			N/A			215 (72.2)				26 (87)			N/A		
**Vital signs**																					
	HR^d^, mean (SD)			90.00 (20.58)			94.38 (21.12)			<.001			102.86 (24.64)				104.73 (23.56)			.68	
	RR^e^, mean (SD)			19.53 (6.44)			20.38 (6.42)			<.001			22.81 (5.97)				23.97 (6.39)			.35	
	SBP^f^, mean (SD)			121.21 (24.40)			122.17 (25.61)			.29			119.34 (28.29)				119.00 (29.08)			.95	
	DBP^g^, mean (SD)			67.30 (18.12)			68.01 (19.08)			.29			66.14 (14.18)				66.00 (14.80)			.96	
	Temperature, mean (SD)			36.81 (0.77)			36.81 (0.84)			.88			37.45 (1.71)				37.40 (2.31)			.91	
	SpO_2_^h^, median (Q1-Q3)			98 (95-100)			98 (95-100)			.005			92 (88-97)				91 (87-98)			.18	
**Severity scoring system**																					
	GCS^i^, median (Q1-Q3)			15 (13-15)			15 (13-15)			.38			15 (12-15)				14 (11-15)			.32	
	SOFA^j^, median (Q1-Q3)			5 (3-8)			5 (3-8)			.04			6 (5-8)				6 (4-8)			.87	
	SAPS^k^ II, mean (SD)			40.31 (14.22)			41.70 (15.31)			.01			39.69 (14.19)				42.43 (15.58)			.36	
**Laboratory data**																					
	WBC^l^, mean (SD)			12.62 (6.81)			13.05 (7.38)			.10			14.13 (6.88)				13.26 (5.50)			.43	
	Hemoglobin, mean (SD)			10.51 (2.24)			10.62 (2.13)			.13			10.87 (2.65)				9.71 (2.64)			.03	
	PLT^m^, mean (SD)			195.42 (100.25)			206.41 (111.67)			.005			189.43 (128.43)				191.00 (103.37)			.94	
	ALT^n^, median (Q1-Q3)			26 (16-52)			29 (17-63)			.03			51 (25-81)				27 (19-53)			.11	
	Creatinine, mean (SD)			1.48 (1.46)			1.41 (1.33)			.12			2.23 (1.82)				2.30 (2.43)			.87	
	Sodium, mean (SD)			137.89 (5.59)			138.43 (5.64)			.007			137.45 (5.17)				138.38 (5.86)			.41	
	Potassium, mean (SD)			4.24 (0.77)			4.14 (0.76)			<.001			4.17 (0.72)				4.28 (0.84)			.49	
	Chloride, mean (SD)			104.04 (6.69)			105.40 (6.80)			<.001			104.44 (7.00)				105.38 (6.74)			.47	
	BUN^o^, median (Q1-Q3)			20 (14-34)			21 (14-35)			.85			14 (9-21)				13 (6-21)			.90	
	Hematocrit, mean (SD)			31.99 (6.67)			32.03 (6.41)			.85			32.29 (9.06)				29.36 (7.93)			.07	
	Glucose, median (Q1-Q3)			130 (106-165)			127 (105-162)			.14			8 (6-11)				8 (6-10)			.90	
	INR^p^, median (Q1-Q3)			1.3 (1.2-1.6)			1.3 (1.2-1.5)			.81			1.3 (1.2-1.5)				1.2 (1.1-1.6)			.42	
	PT^q^, median (Q1-Q3)			14.6 (12.8-17.0)			14.6 (13.0-17.1)			.95			15.7 (14.4-17.6)				14.9 (14.2-16.8)			1.00	
	PTT^r^, median (Q1-Q3)			31.1 (27.3-37.6)			32.2 (27.7-41.6)			<.001			31.9 (27.7-38.2)				39.9 (35.1-46.8)			.17	
	pH^s^, mean (SD)			7.36 (0.10)			7.37 (0.10)			.15			7.40 (0.10)				7.37 (0.11)			.33	
	PaO_2_^t^, median (Q1-Q3)			140.0 (87.0-273.0)			129.0 (84.0-239.5)			.006			93.5 (74.3-121.0)				85.5 (67.0-112.0)			.17	
	PaCO_2_^u^, mean (SD)			42.35 (12.06)			41.04 (12.52)			.003			38.51 (13.15)				39.59 (17.02)			.74	
	BE^v^, mean (SD)			–1.43 (5.24)			–1.47 (5.44)			.82			–2.05 (5.50)				–2.76 (5.31)			.49	
	Lac^w^, median (Q1-Q3)			1.8 (1.2-2.7)			1.8 (1.2-2.6)			.55			2.3 (1.5-3.5)				2.0 (1.5-3.0)			.82	
	HCO_3_^-x^, mean (SD)			22.62 (4.76)			23.12 (4.81)			.003			22.76 (5.11)				22.27 (5.11)			.62	
**Ventilation Variables, median (Q1-Q3)**																					
	PEEP^y^			5 (5-5)			5 (5-5)			.14			3 (2-5)				5 (5-5)			.001	
	FiO_2_^z^			60 (50-100)			60 (50-100)			.09			40 (33-500)				40 (33-50)			.82	
	PEEP^y^/FiO_2_^z^			247 (153-352)			245 (146-345)			.52			230 (168-307)				203 (152-295)			.24	
**Comorbidities, n (%)**																					
	Hypertension			10083 (41.4)			374 (44.3)			.09			214 (71.8)				21 (70)			.83	
	Diabetes			3521 (14.5)			0 (0.0)			<.001			85 (28.5)				5 (17)			.17	
	Myocardial infarction			1085 (4.5)			11 (1.3)			<.001			43 (14.4)				4 (13)			.87	
	COPD^aa^			1647 (6.8)			0 (0.0)			<.001			40 (13.4)				3 (10)			.60	
	Asthma			1912 (7.9)			78 (9.2)			.14			19 (6.4)				0 (0)			.15	
	Pancreatitis			600 (2.5)			38 (4.5)			<.001			10 (3.4)				4 (13)			.01	
	ARF^bb^			8202 (33.7)			339 (40.2)			<.001			180 (60.4)				24 (80)			.04	
	AKI^cc^			16710 (68.6)			652 (77.3)			<.001			187 (62.8)				16 (53)			.31	
	ARDS^dd^			5604 (23.0)			260 (30.8)			<.001			54 (18.1)				16 (53)			<.001	
	Malignant cancer			3829 (15.7)			152 (18.0)			.07			64 (21.5)				8 (27)			.51	
**Therapeutic interventions, n (%)**																					
	Arterial catheterization			1595 (6.6)			166 (19.7)			<.001			55 (18.5)				30 (100)			<.001	
	Central venous catheterization			2028 (8.3)			218 (25.8)			<.001			45 (15.1)				27 (90)			<.001	
	CPB^ee^			2888 (11.9)			77 (9.1)			.02			30 (10.1)				1 (3)			.23	
	CRRT^ff^			2175 (8.9)			96 (11.4)			.02			30 (10.1)				10 (33)			<.001	
	Ventilation			12004 (49.3)			416 (49.3)			1.00			148 (49.7)				25 (83)			<.001	
	Heparin			21304 (87.5)			786 (93.1)			<.001			254 (85.2)				29 (97)			.08	
	Aspirin			12522 (51.4)			384 (45.5)			<.001			135 (45.3)				7 (23)			.02	
	Vasopressors			7538 (31.0)			290 (34.4)			.04			107 (35.9)				25 (83)			<.001	

^a^MIMIC: Medical Information Mart for Intensive Care Unit.

^b^VTE: venous thromboembolism.

^c^N/A: not applicable.

^d^HR: heart rate.

^e^RR: respiratory rate.

^f^SBP: systolic blood pressure.

^g^DBP: diastolic blood pressure.

^h^SpO_2_: oxygen saturation.

^i^GCS: Glasgow Coma Scale.

^j^SOFA: Sequential Organ Failure Assessment.

^k^SAPS II: simplified acute physiology score II.

^l^WBC: white blood cell count.

^m^PLT: platelet.

^n^ALT: alanine aminotransferase.

^o^BUN: blood urea nitrogen.

^p^INR: international normalized ratio.

^q^PT: prothrombin time.

^r^PTT: partial thromboplastin time.

^s^pH: potential of hydrogen.

^t^PaO_2_: partial pressure of oxygen.

^u^PaCO_2_: partial pressure of carbon dioxide.

^v^BE: base excess.

^w^Lac: lactic acid.

^x^HCO_3_^--^: bicarbonate.

^y^PEEP: positive end-expiratory pressure.

^z^FiO_2_: fraction of inspired oxygen.

^aa^COPD: chronic obstructive pulmonary disease.

^bb^ARF: acute respiratory failure.

^cc^AKI: acute kidney injury.

^dd^ARDS: acute respiratory distress syndrome.

^ee^CPB: cardiopulmonary bypass.

^ff^CRRT: continuous renal replacement therapy.

### Selection of Predictors

**Figure 2:**
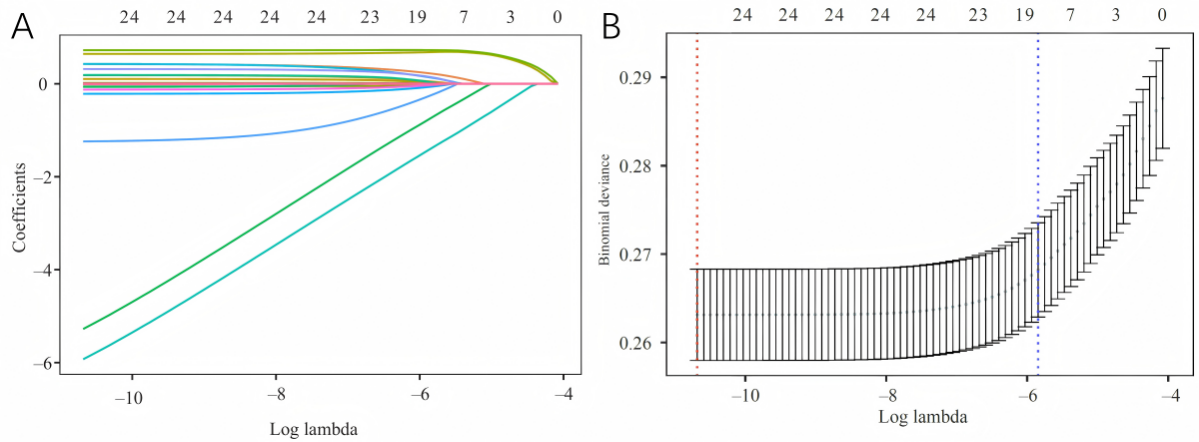
Screening predictors of venous thromboembolism using least absolute shrinkage and selection operator regression. (A) Coefficient profile plotted against the logarithm of the lambda sequence. Each colored line represents the coefficient path of an individual candidate predictor. (B) Cross-validation plot for determining the optimal penalty term. The green dots represent the mean binomial deviance with error bars. The left red vertical dashed line indicates the log lambda value that minimizes the deviance (log lambda minimum = –10.683), and the right blue vertical dashed line indicates the log lambda value within 1 standard error of the minimum (log lambda 1se = –5.845).

Patients in the training set were divided into VTE and non-VTE groups. Univariate analysis identified several variables significantly associated with VTE, including admission age, insurance, HR, RR, SAPS II, alanine aminotransferase, potassium, chloride, glucose, PTT, PaO_2_, PaCO_2_, PEEP, and comorbidities such as diabetes, MI, COPD, pancreatitis, ARF, AKI, and ARDS (all *P*<.05). In addition, therapeutic interventions including arterial catheterization, central venous catheterization, cardiopulmonary bypass, CRRT, heparin use, and aspirin use were significantly associated with VTE (all *P*<.05; Table 2). Least absolute shrinkage and selection operator regression identified 18 variables with nonzero coefficients at the optimal lambda value of 0.0029. These included age, insurance, HR, potassium, chloride, PTT, diabetes, MI, COPD, pancreatitis, ARF, AKI, ARDS, arterial catheterization, central venous catheterization, CRRT, heparin use, and aspirin use (Figures 2A and 2B). These variables were subsequently entered into a multivariate LR model, which revealed that potassium (OR 0.8711, 95% CI 0.7541-0.9756; *P*=.02), chloride (OR 1.0206, 95% CI 1.0081-1.0326; *P*=.001), PTT (OR 1.0086, 95% CI 1.0054-1.0115; *P*<.001), MI (OR 0.2851, 95% CI 0.1101-0.5999; *P*=.003), AKI (OR 1.5274, 95% CI 1.2166-1.9369; *P*<.001), arterial catheterization (OR 1.8985, 95% CI 1.4102-2.5648; *P*<.001), central venous catheterization (OR 2.0805, 95% CI 1.5213-2.8382; *P*<.001), and heparin use (OR 1.5509, 95% CI 1.1203-2.0851; *P*=.01) were independently associated with VTE risk (Table 3). We noted instances of complete separation in the training set for comorbidities such as diabetes and COPD, where the VTE subgroup contained zero cases, leading to extreme ORs in the multivariable LR.

**Table 2:** Univariate analysis of the clinical features in the training set.

Variables				Total (N=17,637)		Non-VTE^a^ (n=17,061)		VTE (n=576)		*P* value
**Demographic data**										
	Age (years), mean (SD)		66.49 (16.30)		66.57 (16.27)		64.10 (16.93)		<.001	
	Sex (male), n (%)		10268 (58.2)		9934 (58.2)		334 (58.0)		.91	
	BMI, mean (SD)		27.75 (6.20)		27.74 (6.20)		27.90 (6.04)		.53	
	**Race, n (%)**								.42	
		Asian	1415 (8.0)		1360 (8.0)		55 (9.6)			
		Black	11463 (65.0)		11,085 (65.0)		378 (65.6)			
		White	604 (3.4)		583 (3.4)		21 (3.7)			
		Hispanic	3642 (20.7)		3538 (20.7)		104 (18.1)			
		Other	513 (2.9)		495 (2.9)		18 (3.1)			
	**Insurance, n (%)**								.004	
		Other	2937 (16.7)		2816 (16.5)		121 (21.0)			
		Medicare	14700 (83.4)		14245 (83.5)		455 (79.0)			
**Vital signs**										
	HR^b^, mean (SD)		90.17 (20.61)		90.06 (20.57)		93.50 (21.61)		<.001	
	RR^c^, mean (SD)		19.52 (6.47)		19.50 (6.46)		20.18 (6.59)		.02	
	SBP^d^, mean (SD)		121.14 (24.40)		121.13 (24.38)		121.45 (25.14)		.77	
	DBP^e^, mean (SD)		67.35 (18.17)		67.34 (18.14)		67.48 (19.10)		.86	
	Temperature, mean (SD)		36.81 (0.77)		36.81 (0.77)		36.74 (0.84)		.06	
	SpO_2_^f^, median (Q1-Q3)		98 (95-100)		98 (95-100)		98 (95-100)		.07	
**Severity scoring system**										
	GCS^g^, median (Q1-Q3)		15 (13-15)		15 (13-15)		15 (13-15)		.67	
	SOFA^h^, median (Q1-Q3)		5 (3-8)		5 (3-8)		5 (4-8)		.12	
	SAPS^i^ II, mean (SD)		40 (14)		40 (14)		42 (16)		.01	
**Laboratory data**										
	WBC^j^, mean (SD)		12.65 (6.84)		12.64 (6.82)		13.04 (7.35)		.19	
	Hemoglobin, mean (SD)		10.51 (2.25)		10.50 (2.25)		10.58 (2.11)		.42	
	PLT^k^, mean (SD)		195.34 (100.17)		195.06 (99.70)		203.51 (113.05)		.08	
	ALT^l^, median (Q1-Q3)		26 (16-53)		26 (16-53)		30 (17-62)		<.001	
	Creatinine, mean (SD)		1.48 (1.45)		1.48 (1.46)		1.43 (1.28)		.39	
	Sodium, mean (SD)		137.90 (5.62)		137.89 (5.62)		138.25 (5.65)		.13	
	Potassium, mean (SD)		4.24 (0.77)		4.24 (0.77)		4.12 (0.73)		<.001	
	Chloride, mean (SD)		104.10 (6.68)		104.06 (6.68)		105.29 (6.73)		<.001	
	BUN^m^, median (Q1-Q3)		20 (14-34)		20 (14-34)		22 (14-35)		.20	
	Hematocrit, mean (SD)		31.98 (6.71)		31.98 (6.72)		31.87 (6.37)		.67	
	Glucose, median (Q1-Q3)		130 (106-165)		130 (106-165)		125 (103-159)		.03	
	INR^n^, median (Q1-Q3)		1.3 (1.2-1.6)		1.3 (1.2-1.6)		1.3 (1.2-1.5)		.54	
	PT^o^, median (Q1-Q3)		14.6 (12.8-17.0)		14.6 (12.8-16.9)		14.6 (13.1-17.1)		.35	
	PTT^p^, median (Q1-Q3)		31.2 (27.3-37.6)		31.2 (27.3-37.6)		32.6 (27.9-42.2)		<.001	
	pH^q^, mean (SD)		7.36 (0.10)		7.36 (0.10)		7.36 (0.10)		.37	
	PaO_2_^r^, median (Q1-Q3)		140 (87-273)		141 (87-275)		129 (86-243)		.04	
	PaCO_2_^s^, mean (SD)		42.32 (12.07)		42.36 (12.04)		41.14 (13.06)		.03	
	BE^t^, mean (SD)		–1.47 (5.26)		–1.47 (5.25)		–1.51 (5.58)		.87	
	Lac^u^, median (Q1-Q3)		1.8 (1.2-2.7)		1.8 (1.2-2.7)		1.8 (1.2-2.7)		.63	
	HCO_3_^-v^, mean (SD)		22.58 (4.76)		22.57 (4.75)		22.87 (4.82)		.14	
**Ventilation Variables, median (Q1-Q3)**										
	PEEP^w^		5 (5-5)		5 (5-5)		5 (5-5)		.045	
	FiO_2_^x^		60 (50-100)		60 (50-100)		60 (50-100)		.13	
	PEEP^w^/FiO_2_^x^		246 (152-353)		246 (152-353)		254 (149-357)		.84	
**Comorbidities, n (%)**										
	Hypertension		7333 (41.6)		7075 (41.5)		258 (44.8)		.11	
	Diabetes		2463 (14.0)		2463 (14.4)		0 (0.0)		<.001	
	Myocardial infarction		793 (4.5)		787 (4.6)		6 (1.0)		<.001	
	COPD^y^		1142 (6.5)		1142 (6.7)		0 (0.0)		<.001	
	Asthma		1381 (7.8)		1327 (7.8)		54 (9.4)		.16	
	Pancreatitis		449 (2.6)		421 (2.5)		28 (4.9)		<.001	
	ARF^z^		5965 (33.8)		5727 (33.6)		238 (41.3)		<.001	
	AKI^aa^		12163 (69.0)		11,712 (68.7)		451 (78.3)		<.001	
	ARDS^bb^		4117 (23.3)		3943 (23.1)		174 (30.2)		<.001	
	Malignant cancer		2790 (15.8)		2685 (15.7)		105 (18.2)		.11	
**Therapeutic interventions, n (%)**										
	Arterial catheterization		1265 (7.2)		1150 (6.7)		115 (20.0)		<.001	
	Central venous catheterization		1580 (9.0)		1443 (8.5)		137 (23.8)		<.001	
	CPB^cc^		2090 (11.9)		2038 (12.0)		52 (9.0)		.03	
	CRRT^dd^		1605 (9.1)		1533 (9.0)		72 (12.5)		.004	
	Ventilation		8718 (49.4)		8442 (49.5)		276 (47.9)		.46	
	Heparin		15479 (87.8)		14943 (87.6)		536 (93.1)		<.001	
	Aspirin		9026 (51.2)		8771 (51.4)		255 (44.3)		<.001	
	Vasopressors		5492 (31.1)		5294 (31.0)		198 (34.4)		.09	

^a^VTE: venous thromboembolism.

^b^HR: heart rate.

^c^RR: respiratory rate.

^d^SBP: systolic blood pressure.

^e^DBP: diastolic blood pressure.

^f^SpO_2_: oxygen saturation.

^g^GCS: Glasgow Coma Scale.

^h^SOFA: Sequential Organ Failure Assessment.

^i^SAPS II: simplified acute physiology score II.

^j^WBC: white blood cell count.

^k^PLT: platelet.

^l^ALT: alanine aminotransferase.

^m^BUN: blood urea nitrogen.

^n^INR: international normalized ratio.

^o^PT: prothrombin time.

^p^PTT: partial thromboplastin time.

^q^pH: potential of hydrogen.

^r^PaO_2_: partial pressure of oxygen.

^s^PaCO_2_: partial pressure of carbon dioxide.

^t^BE: base excess.

^u^Lac: lactic acid.

^v^HCO_3_^-^: bicarbonate.

^w^PEEP: positive end-expiratory pressure.

^x^FiO_2_: fraction of inspired oxygen.

^y^COPD: chronic obstructive pulmonary disease.

^z^ARF: acute respiratory failure.

^aa^AKI: acute kidney injury.

^bb^ARDS: acute respiratory distress syndrome.

^cc^CPB: cardiopulmonary bypass.

^dd^CRRT: continuous renal replacement therapy.

**Table 3:** Multivariate logistic regression analysis of the selected clinical features in the training set.

Variables	β	*Z* value	Odds ratio	95% CI	*P* value
Admission age (years)	–.0018	–0.6384	0.9982	–0.0072 to 0.0037	.52
Insurance	–.2206	–1.9143	0.8020	–0.4436 to 0.0084	.06
HR^a^	.0035	1.6880	1.0035	–0.0006 to 0.0076	.09
Potassium	–.1380	–2.3558	0.8711	–0.2541 to –0.0244	.02
Chloride	.0204	3.2561	1.0206	0.0081 to 0.0326	.001
PTT^b^	.0085	5.5201	1.0086	0.0054 to 0.0115	<.001
Diabetes	–15.8672	–0.0766	0.0000	–162.9076 to –157.5869	.94
Myocardial infarction	–1.2549	–2.9781	0.2851	–2.1976 to –0.5169	.003
COPD^c^	–15.5638	–0.0530	0.0000	–222.8419 to –215.7841	.96
Pancreatitis	.3493	1.6805	1.4181	–0.0786 to 0.7391	.09
ARF^d^	.1127	1.2010	1.1192	–0.0721 to 0.2957	.23
AKI^e^	.4236	3.9539	1.5274	0.2166 to 0.6369	<.001
ARDS^f^	.1806	1.8616	1.1979	–0.0116 to 0.3689	.06
Arterial catheterization	.6411	5.5322	1.8985	0.4102 to 0.8648	<.001
Central venous catheterization	.7326	6.8935	2.0805	0.5213 to 0.9382	<.001
CRRT^g^	.2571	1.8355	1.2931	–0.0240 to 0.5257	.07
Heparin	.4388	2.5944	1.5509	0.1203 to 0.7851	.01
Aspirin	–.0882	–0.9590	0.9156	–0.2687 to 0.0917	.34

^a^HR: heart rate.

^b^PTT: partial thromboplastin time.

^c^COPD: chronic obstructive pulmonary disease.

^d^ARF: acute respiratory failure.

^e^AKI: acute kidney injury.

^f^ARDS: acute respiratory distress syndrome.

^g^CRRT: continuous renal replacement therapy.

### Development and Validation of Models

**Figure 3:**
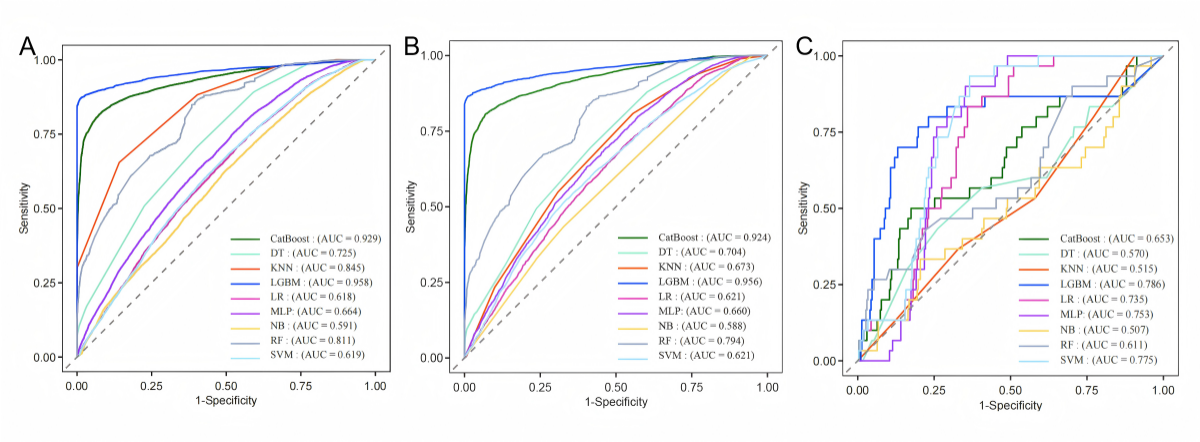
Receiver operating characteristic (ROC) curves of the test and validation sets of 9 machine learning models. (A) ROC curves of the training set. (B) ROC curves of the internal validation set. (C) ROC curves of the external validation set. AUC: area under the curve; CatBoost: categorical boosting; DT: decision tree; KNN: k-nearest neighbor; LGBM: light gradient boosting machine; LR: logistic regression; MLP: multilayer perceptron; NB: naive Bayes; RF: random forest; SVM: support vector machine.

All selected variables were incorporated into the development of 9 ML models to predict the risk of VTE in ICU patients with sepsis. The 9 ML models exhibited varying degrees of predictive performance in the training set (Figure 3A). Internal validation based on receiver operating characteristic curve analysis demonstrated that the LGBM model achieved the highest predictive performance (AUC=0.956), followed by CatBoost (AUC=0.924), RF (AUC=0.794), decision tree (AUC=0.704), k-nearest neighbors (AUC=0.673), multilayer perceptron (AUC=0.660), support vector machine (AUC=0.621), LR (AUC=0.621), and naïve Bayes (AUC=0.588; Figure 3B). Detailed performance metrics, including sensitivity, specificity, accuracy, precision, *F*_1_-score, and AUC for each model, are shown in Table 4. Notably, the LGBM model exhibited the highest specificity (0.942), accuracy (0.910), precision (0.956), and *F*_1_-score (0.921), consistently outperforming the other algorithms across these key evaluation indicators.

**Table 4:** Comprehensive evaluation of machine learning model performance.

Methods	Sensitivity	Specificity	Accuracy	Precision	*F*_1_	AUC^a^
CatBoost^b^	0.926	0.598	0.791	0.766	0.839	0.924
DT^c^	0.881	0.394	0.680	0.675	0.764	0.704
KNN^d^	0.809	0.444	0.658	0.674	0.735	0.673
LGBM^e^	0.888	0.942	0.910	0.956	0.921	0.956
LR^f^	0.938	0.199	0.633	0.625	0.750	0.621
MLP^g^	0.803	0.504	0.680	0.698	0.747	0.660
NB^h^	0.308	0.769	0.498	0.655	0.419	0.588
RF^i^	0.893	0.438	0.705	0.694	0.781	0.794
SVM^j^	0.991	0.027	0.594	0.592	0.741	0.621

^a^AUC: area under the curve.

^b^CatBoost: categorical boosting.

^c^DT: decision tree.

^d^KNN: k-nearest neighbor.

^e^LGBM: light gradient boosting machine.

^f^LR: logistic regression.

^g^MLP: multilayer perceptron.

^h^NB: naive Bayes.

^i^RF: random forest.

^j^SVM: support vector machine.

**Figure 4:**
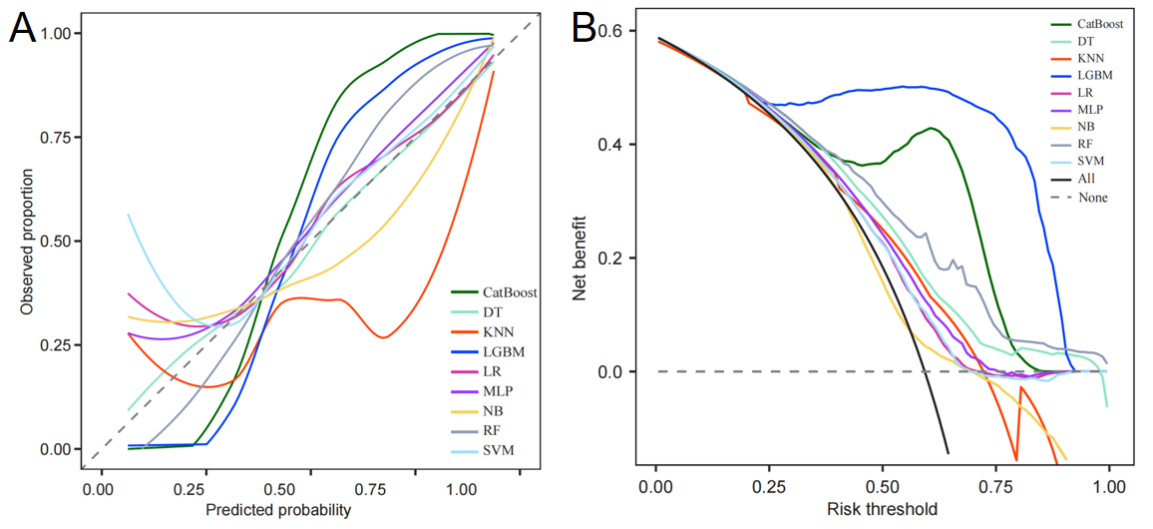
Calibration curve of the LGBM model in the internal validation set (A). Decision curve analysis of the LGBM model in the internal validation set (B). CatBoost: categorical boosting; DT: decision tree; KNN: k-nearest neighbor; LGBM: light gradient boosting machine; LR: logistic regression; MLP: multilayer perceptron; NB: naive Bayes; RF: random forest; SVM: support vector machine.

Calibration analysis revealed that the LGBM model demonstrated strong alignment between predicted probabilities and observed outcomes, reflecting robust reliability (Figure 4A). In the decision curve analysis, the LGBM model consistently provided the highest net benefit across a broad range of clinically relevant threshold probabilities, further supporting its clinical applicability in decision-making scenarios (Figure 4B).

External validation using an independent test cohort further confirmed the robustness and generalizability of the LGBM model, which achieved an AUC of 0.786, outperforming all other ML models in this cohort (Figure 3C). The consistent performance observed across both internal and external validation cohorts underscores the strong generalizability and clinical potential of the LGBM model for predicting VTE in ICU patients with sepsis.

Following stratification based on SOFA score, the LGBM model demonstrated enhanced discriminative ability in the high-risk group. The AUC for the severe sepsis group was 0.816, which was higher than the AUC of 0.769 observed in the mild to moderate sepsis group. The detailed performance metrics are shown in Table 5.

**Table 5:** Subgroup analysis of LGBM^a^ model performance stratified by SOFA^b^ score.

Subgroup	SOFA Criteria	Sensitivity	Specificity	AUC^c^
Mild to moderate sepsis	2 ≤ SOFA ≤ 6	0.722	0.839	0.769
Severe sepsis	SOFA > 6	0.667	0.856	0.816

^a^LGBM: light gradient boosting machine.

^b^SOFA: Sequential Organ Failure Assessment.

^c^AUC: area under the curve.

### Explanation of the Model

**Figure 5:**
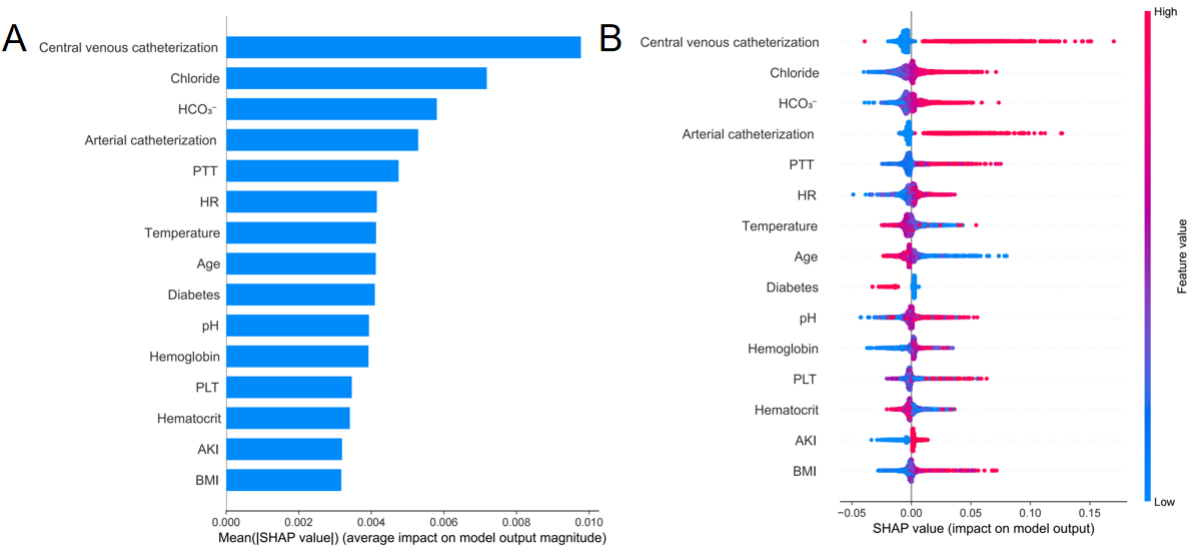
SHAP summary plot for clinical variables contributing to the light gradient boosting machine (LGBM) model. (A) Feature importance ranking plot based on the LGBM model. (B) Scatter plot of variables for SHAP analysis based on the LGBM model. AKI: acute kidney injury; HCO3-: bicarbonate; HR: heart rate; pH: potential of hydrogen; PLT: platelet; PTT: partial thromboplastin time; SHAP: SHapley Additive exPlanations.

Figure 5 displays the top 15 predictive features for VTE incidence as identified by the LGBM model using SHAP analysis. The feature importance plot, ranked by the mean absolute SHAP values, highlights the variables with the greatest overall impact on model predictions (Figure 5A). The 5 most influential features were central venous catheterization, chloride, bicarbonate, arterial catheterization, and PTT. The corresponding SHAP beeswarm plot (Figure 5B) illustrates each feature’s influence on individual predictions, where SHAP values to the right of zero reflect increased predicted risk. It revealed that the presence of central venous or arterial catheters, elevated chloride and bicarbonate levels, and prolonged PTT consistently contributed to a higher VTE risk.

**Figure 6:**
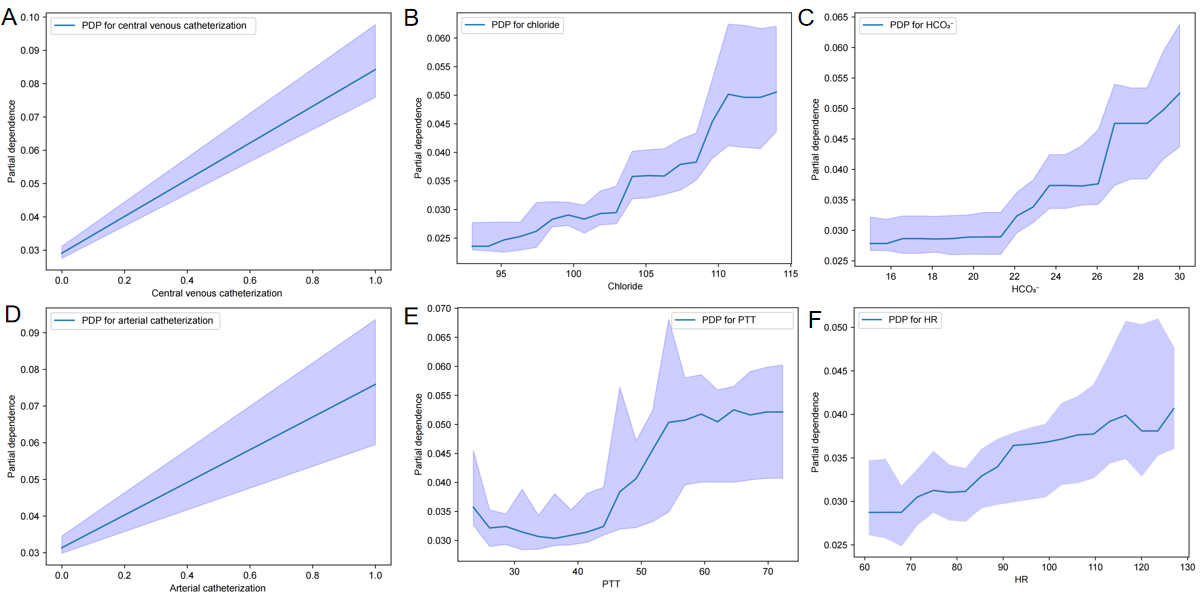
SHapley Additive exPlanations feature PDP of the light gradient boosting machine model. (A) Partial dependence plot of central venous catheter. (B) Partial dependence plot of chloride. (C) Partial dependence plot of bicarbonate. (D) Partial dependence plot of arterial catheter. (E) Partial dependence plot of partial thromboplastin time. (F) Partial dependence plot of heart rate. HCO3-: bicarbonate; HR: heart rate; PDP: partial dependence plot; PTT: partial thromboplastin time.

To further examine the marginal effects of key predictors on model output, PDPs were generated for the top-ranked features (Figures 6A-6F). These plots depict how the predicted risk of VTE changes on average with varying values of a single feature, while holding others constant. For dichotomous variables such as central venous (Figure 6A) and arterial (Figure 6D) catheterization, the plots showed a marked increase in the average predicted risk when catheters were present compared with their absence. Continuous variables including serum chloride (Figure 6B), bicarbonate (Figure 6C), and HR (Figure 6F) exhibited monotonic relationships, with risk increasing steadily as the values rose. In contrast, PTT (Figure 6E) exhibited a distinct threshold effect. The predicted risk remained low and stable within the normal range and then increased sharply for values beyond approximately 45 seconds before plateauing at highly prolonged levels, confirming its nonlinear risk association identified by SHAP.

### Interpretation of Individual Prediction

**Figure 7:**
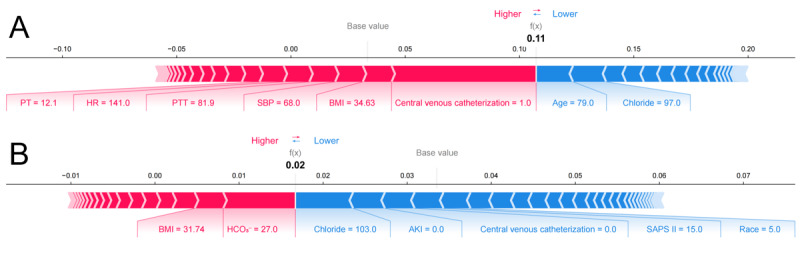
SHapley Additive exPlanations (SHAP) force plots for explaining of individual’s prediction results according to the light gradient boosting machine model. (A) SHAP force plot of a high-risk example. (B) SHAP force plot of a low-risk example. AKI: acute kidney injury; HCO3-: bicarbonate; HR: heart rate; PT: prothrombin time; PTT: partial thromboplastin time; SAPS: simplified acute physiology score II; SBP: systolic blood pressure.

To enhance case-level interpretability, SHAP force plots were used to visualize the contribution of individual features to specific predictions (Figure 7). In these plots, each feature was represented as a colored bar that moves the predicted value away from a baseline, which corresponds to the average prediction across the dataset. Features that increase the predicted risk are shown in red, while those that reduce the risk are shown in blue. The length of each bar reflects the extent of the feature’s influence. A longer bar indicates a stronger impact on the final predicted probability.

For example, in Figure 7A, the predicted VTE probability for a specific patient was 0.11, which was higher than the baseline value. This elevated risk was primarily attributed to the presence of a central venous catheter, a markedly prolonged PTT of 81.9 seconds, and a high HR of 141 min^−1^. In contrast, Figure 7B showed a patient with a predicted probability of 0.02, which was lower than the baseline. In this case, protective features including the absence of a central venous catheter, absence of AKI, and a low SAPS II score collectively outweighed the influence of risk factors such as elevated BMI and bicarbonate level. These examples demonstrated that the model was capable of producing clear and clinically relevant explanations for individual predictions.

## Discussion

### Principal Findings

In this multicenter retrospective study, we developed and validated several ML models to predict the risk of VTE in critically ill patients with sepsis. Among the models tested, the LGBM algorithm demonstrated the highest predictive accuracy with an AUC of 0.956 in the internal validation set and maintained strong generalizability with an AUC of 0.786 in an external cohort. The observed VTE incidence in our external validation cohort (30/328, 9.15%) was substantially higher than the 3.35% (844/25,197) incidence recorded in the MIMIC-IV development cohort. While this difference contributed to the drop in the AUC during external validation, this discrepancy is likely a consequence of multiple factors, including divergent diagnostic practices, disparities in clinical risk profiles, and racial composition differences between the 2 cohorts.

First, variations in VTE surveillance and diagnostic criteria likely served as a primary driver. The MIMIC-IV cohort relied on retrospective identification via *ICD* (*International Classification of Diseases*) codes, a method known to carry a risk of misclassification bias and potential underascertainment, particularly of asymptomatic events. Conversely, the external validation cohort used explicit diagnostic imaging reports and implemented a routine lower limb venous ultrasound screening program for high-risk patients with sepsis. This systematic, active surveillance methodology inherently tends to yield a higher VTE capture rate compared with passive, coding-based case ascertainment. Second, the external validation group exhibited a substantially higher baseline severity and utilization of aggressive life support interventions. Patients with VTE in this cohort showed markedly higher rates of established risk factors such as mechanical ventilation (83.33% vs 49.29% in MIMIC-IV VTE group), ARDS (53.33% vs 30.81%), and CRRT (33.33% vs 11.37%) [19-21]. This heightened clinical severity and iatrogenic risk profile provide a sound clinical explanation for the elevated VTE incidence. Third, the inherent demographic differences between the cohorts represent a major challenge to model generalizability. The MIMIC-IV cohort is composed of a racially diverse population, while the external validation cohort is predominantly Asian, representing a geographically and ethnically distinct cohort. Given that VTE risk is known to vary significantly across different racial groups, this change in demographic distribution may be a contributing factor to the observed performance decrease [22].

Despite these significant differences in cohort characteristics and VTE incidence, the LGBM model maintained the highest predictive performance in both internal and external validation, underscoring its inherent robustness and generalization capacity. These performance advantages align with recent findings that gradient boosting methods often outperform logistic models in predictive accuracy when applied to large clinical datasets [23,24]. Crucially, we applied SHAP to interpret the model’s predictions. This analysis identified a set of key risk factors driving VTE predictions, including the presence of central and arterial catheters, serum chloride and bicarbonate levels, and the PTT. By quantifying each feature’s contribution to individual risk scores, SHAP facilitated insight into why certain patients were classified as high-risk. This combination of robust performance and interpretability suggests that our model may assist clinicians in identifying ICU patients with sepsis at an increased risk of VTE, thereby supporting timely prophylaxis or diagnostic evaluation. Conventional instruments such as the Caprini, Padua, or Wells scores were primarily developed for general medical or surgical populations and relied on a linear, additive combination of risk factors [10,25]. These tools lack the specificity required to capture the distinct pathophysiology of sepsis which involves a complex interplay among inflammation, endothelial dysfunction, and coagulopathy collectively referred to as sepsis-induced coagulopathy [26,27]. The pathophysiology of sepsis-induced VTE is inherently nonlinear. Complex interactions among organ failure, systemic inflammation, and iatrogenic factors such as the use of catheters and vasopressors jointly modulate VTE risk in a manner that traditional linear models cannot fully capture [28,29]. In contrast, our LGBM model is consistent with previous ML applications in critical care and automatically captures complex relationships and interactions among predictors while integrating heterogeneous ICU data to generate a more accurate and nuanced risk estimate [30]. Our study found that the LGBM model significantly outperformed the LR model, achieving an AUC value of 0.956 compared with 0.621, which underscores the advantage of advanced ML algorithms in modeling the complex relationships among multiple risk factors. We recognize that prior studies have used advanced ML models for VTE prediction in critically ill patients. For instance, Jin et al [31] developed an RF model for VTE prediction in ICU patients that achieved an AUC of 0.788 in their validation dataset. Our model demonstrates equivalent predictive power in a challenging, sepsis-specific cohort. This robust performance likely stems from 2 key strengths of our approach: first, the inherent efficiency and predictive power of the LGBM algorithm compared with other boosting techniques; second, and more critically, our model’s unique inclusion of metabolic and acid-base markers, such as serum chloride and bicarbonate, may offer a more nuanced reflection of the prothrombotic state induced by sepsis-associated organ dysfunction, thereby enhancing predictive accuracy.

The comparative analysis between the LR findings and the SHAP feature ranking reveals the core limitation of traditional statistical approaches in septic VTE prediction. The discrepancy observed for bicarbonate is illustrative. While bicarbonate was not retained in the final LR model, suggesting that its linear signal lacked statistical significance for VTE risk, the LGBM model ranked it as the third most influential predictor overall. This strongly suggests that bicarbonate’s predictive power does not stem from a simple linear effect but from its complex nonlinear relationship or crucial interaction with other factors, reflecting the severity of systemic acid-base disturbances. Similarly, MI appeared as a protective factor in the final multivariable regression (OR 0.2851), but its marginal contribution was deemed less vital by SHAP than core metabolic and iatrogenic factors. This protective association in the linear model was likely influenced by confounding or collinearity among chronic conditions, potentially because patients with a history of MI often receive stricter chronic anticoagulation management, which may suppress their VTE risk and mask the true underlying risk in the linear model. The issue of complete separation for variables such as diabetes and COPD further highlights the inherent defect of LR when processing sparse data, as it led to unreliable, extreme coefficients. These findings collectively validate the choice of the nonlinear LGBM model and underscore the necessity of relying on its transparent SHAP feature analysis to evaluate the true, complex VTE risk drivers that are often obscured by the restrictive linearity of traditional statistical methods. Furthermore, the finding that the LGBM model achieved a higher AUC (0.816 vs 0.769) in the severe sepsis subgroup is significant. This enhanced discriminative power in the highest risk population confirms the model’s clinical use, suggesting that it is particularly effective for targeted VTE prophylaxis and early intervention in patients with multiorgan dysfunction.

The interpretability enabled by SHAP analysis is essential for clinical translation and offers valuable pathophysiological insights. The top predictors identified by our SHAP analysis are closely related to the pathophysiology of sepsis-associated VTE and reinforce the model’s clinical plausibility. The paramount importance of central venous catheterization aligns with Virchow’s triad as the catheter can directly cause endothelial injury and lead to local blood flow stasis, both of which are potent triggers for thrombosis [32]. This finding is consistent with numerous studies highlighting indwelling catheters as a major iatrogenic risk factor for VTE in ICU patients [33,34]. The contribution of arterial catheterization, although less pronounced, further underscores the role of endothelial disruption from invasive devices.

Beyond these established risk factors, the model identified the predictive value of metabolic parameters such as serum chloride and bicarbonate. Elevated serum chloride is frequently associated with metabolic acidosis, which commonly develops in severe sepsis and shock [35]. Acidosis has been shown to enhance PLT aggregation and impair fibrinolysis, thereby promoting a prothrombotic state [36]. The association with bicarbonate likely reflects the severity of systemic acid-base disturbances commonly observed in septic shock. These findings suggest that fluid resuscitation strategies and acid-base management may represent modifiable components of VTE risk. The role of PTT is particularly noteworthy. Although a prolonged PTT is traditionally associated with bleeding risk, in sepsis it may paradoxically indicate consumptive coagulopathy in which widespread microthrombosis rapidly consumes clotting factors and leads to their depletion, a hallmark of disseminated intravascular coagulation [37]. The PDP revealed a nonlinear risk profile, where VTE risk increased markedly once PTT exceeded approximately 45 seconds. This nonlinear relationship would likely be overlooked in traditional linear analyses and highlights the model’s capacity to identify clinically meaningful physiological thresholds. The contribution of HR appears more direct as its PDP showed a monotonic increase in predicted VTE risk with rising HR. Tachycardia is a cardinal manifestation of the systemic inflammatory response and catecholamine surge in sepsis, reflecting physiological stress and a hyperdynamic state that may promote endothelial dysfunction and a prothrombotic environment [38,39].

### Strengths and Limitations

A major strength of our study lies in the integration of rigorous model development with interpretability, which enhances its potential clinical applicability. The model was developed using the large, high-quality public MIMIC-IV database and externally validated it in a geographically and ethnically distinct cohort from our own center. This 2-tiered validation strategy is essential for evaluating model generalizability, which remains a common limitation in single-center ML studies [40]. The decline in AUC from 0.956 to 0.786 during external validation, while still maintaining the best performance among all models, underscores the model’s robust generalizability. This performance gap likely reflects intrinsic differences between the 2 cohorts, such as patient genetics, local clinical practices, VTE screening protocols, and data-recording standards. These findings underscore the critical need for local calibration or model retraining before deploying any predictive model in a new clinical setting. In addition, our use of SHAP enhances model transparency and interpretability rather than producing a purely opaque prediction tool. By generating individualized explanations through force plots, our model provides clinicians with transparent and actionable insights that support clinical decision-making and facilitate integration into routine workflows. Such interpretability is essential for translating ML tools from research settings to real-world clinical practice.

Despite these strengths, our study has several limitations. First, its retrospective design allows for the identification of associations but not the establishment of causality. The reliance on *ICD* codes for VTE diagnosis in the MIMIC-IV database may lead to some misclassification, as coding practices can vary and may not capture all clinically recognized events [41]. Second, we included data from only the first 24 hours of ICU admission. While this time frame is critical for early risk stratification, VTE is a dynamic process, and models incorporating time series data may provide greater predictive accuracy [42]. Third, despite comprehensive data extraction, some potential confounders, such as the specific type and dosage of thromboprophylaxis, patient mobility status, or the specific subtypes of malignancy, were not available. This includes the lack of detailed or consistently documented data on heparin dosage, such as prophylactic versus therapeutic, or duration, which forced us to treat heparin use as a binary variable. Finally, although the use of imputation for missing data is standard practice, it may still introduce a small degree of bias.

Future research should aim to prospectively validate our LGBM model in multicenter interventional trials to confirm its clinical use and impact on patient outcomes. Further refinement of the model may involve incorporating dynamic variables throughout the ICU stay and integrating emerging biomarkers related to coagulopathy and endothelial dysfunction. In addition, exploring federated learning approaches could enable the development of more robust and generalizable models across institutions while preserving patient data privacy [43].

### Conclusions

This study presents a high-performing and interpretable LGBM model for predicting VTE in ICU patients with sepsis by effectively integrating a broad spectrum of clinical and laboratory data. By leveraging SHAP to enhance transparency, the model extends beyond a simple predictive tool to function as a decision support system capable of elucidating complex risk profiles and enabling clinicians to implement more personalized and effective VTE prevention strategies. This work lays the foundation for the adoption of sophisticated yet interpretable artificial intelligence–driven tools aimed at reducing the burden of VTE in this high-risk patient population.

## Data Availability

The datasets and code used in this study are available from the corresponding author on reasonable request.

## Abbreviations

AKI: acute kidney injury
ARDS: acute respiratory distress syndrome
ARF: acute respiratory failure
AUC: area under the curve
CatBoost: categorical boosting
COPD: chronic obstructive pulmonary disease
CRRT: continuous renal replacement therapy
FiO_2_: fraction of inspired oxygen
HR: heart rate
ICD: International Classification of Diseases
ICD-9: International Classification of Diseases, Ninth Revision
ICD-10: International Statistical Classification of Diseases, Tenth Revision
ICU: intensive care unit
LGBM: light gradient boosting machine
LR: logistic regression
MI: myocardial infarction
MIMIC-IV: Medical Information Mart for Intensive Care IV
ML: machine learning
OR: odds ratio
PaCO_2_: partial pressure of carbon dioxide
PaO_2_: partial pressure of oxygen
PDP: partial dependence plot
PE: pulmonary embolism
PEEP: positive end-expiratory pressure
PLT: platelet
PT: prothrombin time
PTT: partial thromboplastin time
RF: random forest
RR: respiratory rate
SAPS II: simplified acute physiology score II
SHAP: Shapley Additive Explanations
SMOTE: Synthetic Minority Oversampling Technique
SOFA: Sequential Organ Failure Assessment
SpO_2_: oxygen saturation
VTE: venous thromboembolism
